# Perforation of the right ventricle and inferior vena cava by bone cement after percutaneous vertebroplasty: A case report

**DOI:** 10.1097/MD.0000000000050420

**Published:** 2026-08-28

**Authors:** Yan Dong, Yongfeng Zhu, Zhikun Fu, Yaping Xu, Xiangli Zhang

**Affiliations:** a Department of Cardiac Surgery, Zhengzhou Seventh People’s Hospital, Zhengzhou, Henan, People’s Republic of China; b Henan Key Laboratory of Cardiac Remodeling and Transplantation, Zhengzhou Seventh People’s Hospital, Zhengzhou, Henan, People’s Republic of China.

**Keywords:** bone cement migration, complications, percutaneous vertebroplasty, postoperative monitoring, right ventricular perforation

## Abstract

**Rationale:**

Percutaneous vertebroplasty (PVP) is widely used to treat vertebral compression fractures. Although it is generally considered a minimally invasive procedure, leakage and systemic migration of polymethylmethacrylate bone cement may rarely occur and can cause potentially fatal cardiovascular complications. Intracardiac bone cement embolism accompanied by perforation of the right ventricle and inferior vena cava (IVC) is particularly uncommon.

**Patient concerns:**

A 70-year-old woman presented with palpitations and perioral numbness 2 months after undergoing PVP for an L4 vertebral compression fracture. Her vital signs were stable, and electrocardiographic findings and cardiac troponin levels were within normal ranges.

**Diagnoses:**

Echocardiography, coronary angiography, and computed tomography revealed linear bone cement fragments extending through the IVC, right atrium, and right ventricle. One intracardiac cement fragment had penetrated the right ventricular wall, leading to a diagnosis of cardiac cement embolism with right ventricular and IVC perforation.

**Interventions:**

Because of the risks of progressive myocardial perforation, cardiac tamponade, and other potentially fatal complications, the patient underwent surgical removal of the bone cement fragments with extracorporeal circulation. Both cement fragments were successfully removed, and the perforated right ventricular wall was repaired.

**Outcomes:**

The patient’s postoperative course was uneventful. Follow-up echocardiography before discharge showed no residual foreign body, pericardial effusion, or structural cardiac abnormality.

**Lessons:**

Intracardiac bone cement embolism should be considered in patients who develop unexplained cardiovascular symptoms after PVP, even when their hemodynamic status, electrocardiographic findings, and cardiac biomarkers are initially normal. Prompt multimodal imaging is essential for identifying the location and extent of cement migration. Timely surgical intervention should be considered when cardiac perforation, structural injury, or cardiac tamponade is suspected.

## 1. Introduction

Percutaneous vertebroplasty (PVP) is a commonly used procedure for treating vertebral compression fractures, particularly in patients with osteoporotic vertebral fractures. Although PVP is considered a minimally invasive treatment with generally favorable outcomes, it can also lead to rare but severe complications.^[1]^ Polymethylmethacrylate bone cement leakage is a well-documented complication of PVP, typically occurring in the paravertebral veins, intervertebral discs, or epidural space.^[2]^ However, systemic embolization of bone cement, especially migration into the cardiovascular system, is rare but can result in life-threatening complications. This report presents a rare but serious complication following PVP, emphasizing the importance of close postoperative monitoring and early intervention to reduce the risk of fatal outcomes.

## 2. Case presentation

### 2.1. Patient baseline risk and clinical context

The patient was a 70-year-old woman who had undergone PVP for an L4 vertebral compression fracture 2 months before presentation. The clinically relevant baseline risk context included advanced age, a recent vertebral augmentation procedure, and the absence of routine postoperative imaging surveillance. Her medical history was notable for severe osteoporosis and grade 2 hypertension. She had no history of diabetes mellitus, coronary artery disease, chronic pulmonary disease, or prior thromboembolism. She was not receiving anticoagulant or antiplatelet therapy. During the previous PVP procedure, 4.5 mL of polymethylmethacrylate bone cement was injected, and no intraoperative cement leakage was observed. Information regarding the viscosity of the bone cement and the injection pressure was unavailable.

At admission, she was hemodynamically stable, with normal blood pressure and respiratory function. Electrocardiography and serum troponin testing showed no evidence of acute myocardial ischemia or injury, and coronary angiography excluded obstructive coronary artery disease. Nevertheless, the delayed onset of cardiovascular symptoms after PVP raised concern for a procedure-related complication.

### 2.2. Clinical course and diagnostic assessment

The patient initially presented to a local hospital with palpitations and perioral numbness. Empirical treatment for suspected coronary artery disease was started. Cardiac ultrasonography subsequently revealed a linear hyperechoic structure extending through the right atrium, right ventricle, and inferior vena cava (IVC), raising suspicion of bone cement migration. The patient was referred to our hospital for further evaluation and management.

**Figure 1.**
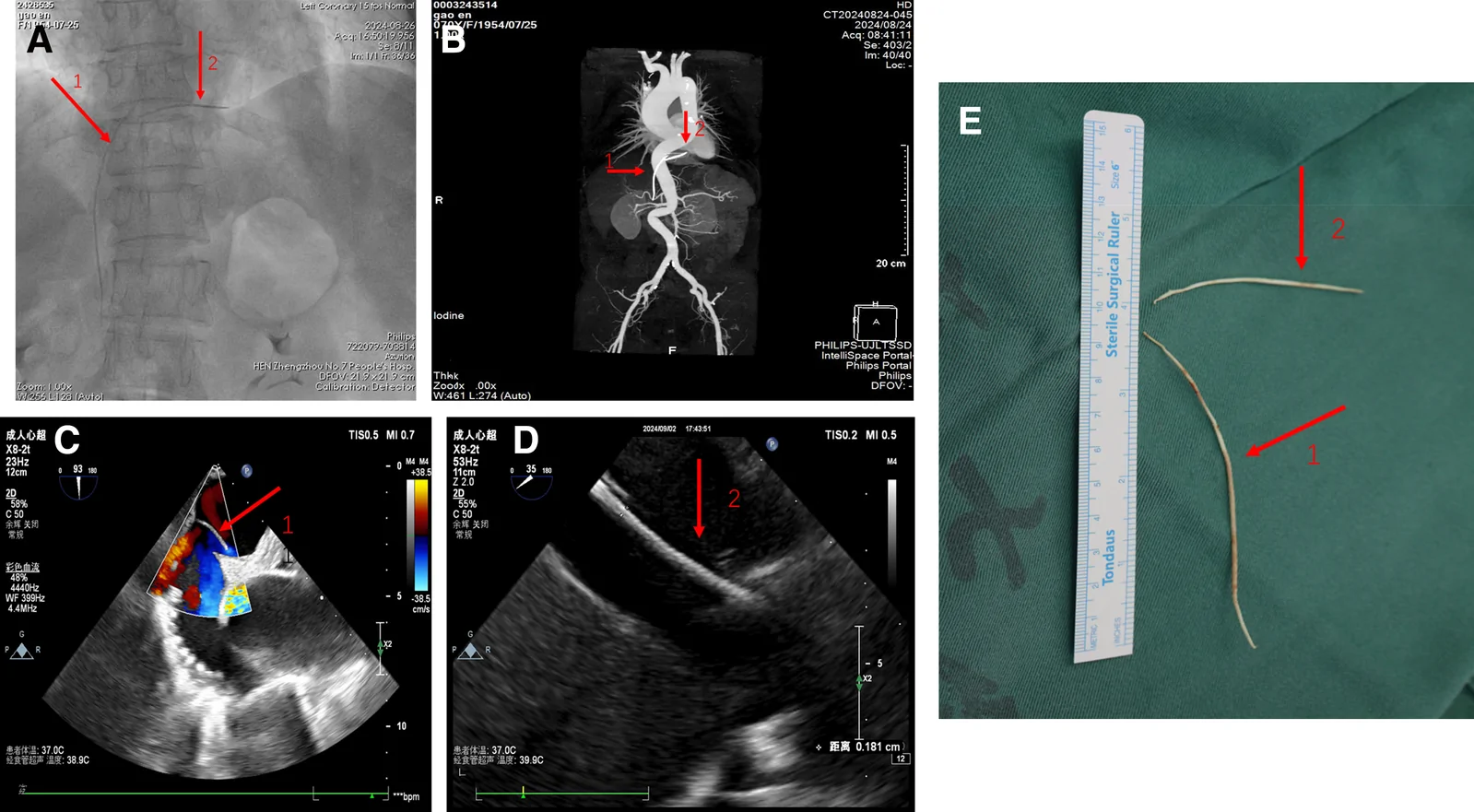
Multimodal imaging and intraoperative findings of intracardiac bone cement embolism. (A) IVC angiography showing bone cement fragments (arrows). (B) CT demonstrating linear high-density bone cement fragments within the IVC and right heart. (C, D) Transesophageal echocardiography showing a linear hyperechoic/striated foreign body extending through the right atrium and right ventricle, without definite pericardial effusion. (E) Intraoperative photograph of the two retrieved bone cement fragments. Arrow 1 indicates the bone cement fragment located at the entrance to the IVC, and arrow 2 indicates the fragment extending from the right atrium into the right ventricle and penetrating the right ventricular wall. Both fragments were removed under extracorporeal circulation, and the right ventricular wall was repaired. CT = computed tomography, IVC = inferior vena cava.

Repeat echocardiography demonstrated a striated foreign body within the right ventricle without definite pericardial effusion. Computed tomography (CT) further confirmed linear high-density foreign bodies in the IVC and right ventricle, consistent with bone cement embolism (Fig. 1). Review of the patient’s history confirmed that no postoperative imaging follow-up had been performed after PVP.

### 2.3. Clinical timeline

**Figure 2.**
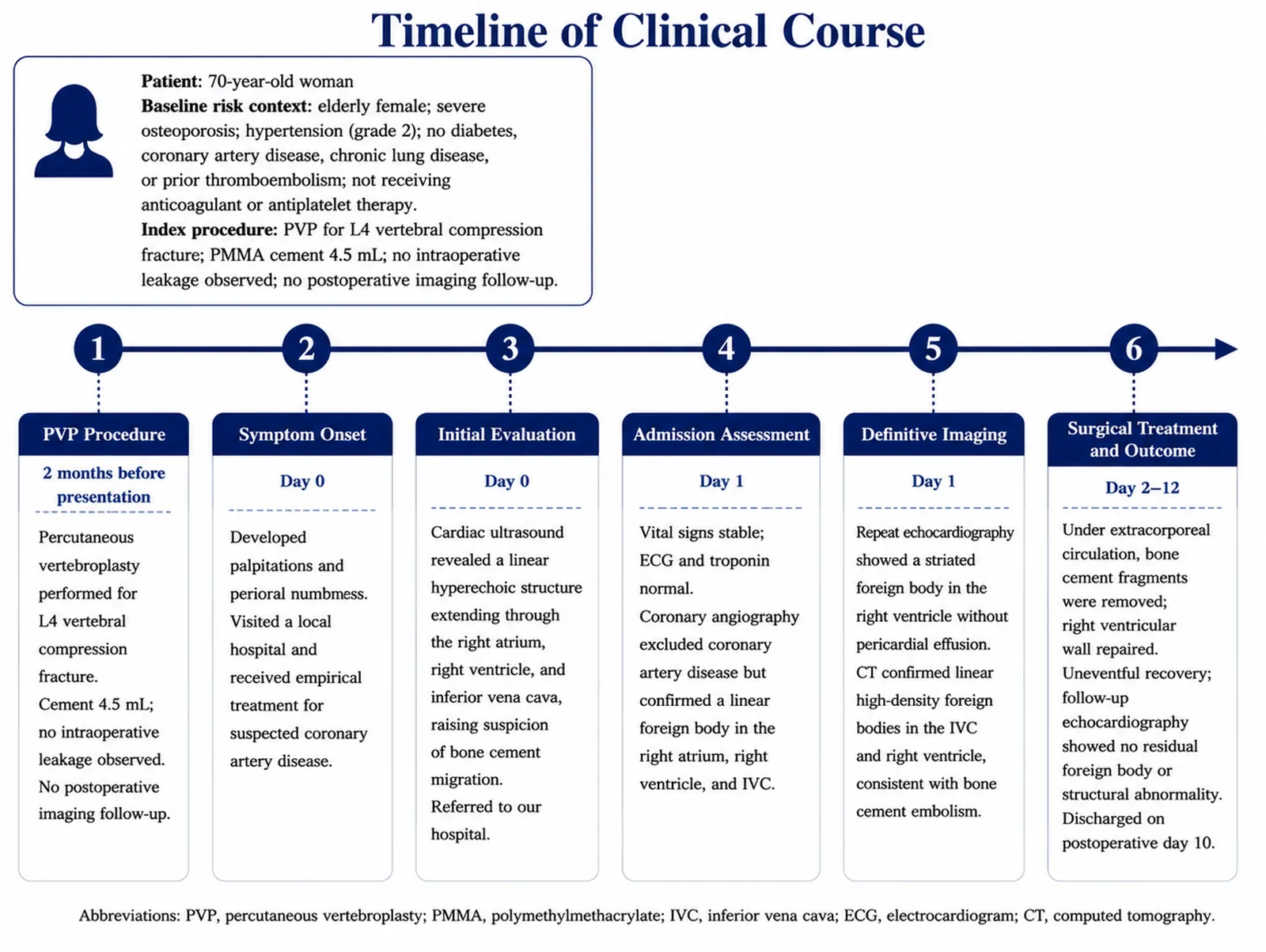
Timeline of the patient’s clinical course from percutaneous vertebroplasty to postoperative recovery. The patient underwent percutaneous vertebroplasty for an L4 vertebral compression fracture 2 months before presentation. On day 0, she developed palpitations and perioral numbness, and cardiac ultrasonography raised suspicion of bone cement migration. After referral to our hospital, admission assessment on day 1 showed stable vital signs, normal electrocardiographic findings and troponin levels, and no obstructive coronary artery disease. Repeat echocardiography and computed tomography confirmed bone cement fragments within the inferior vena cava and right ventricle. Surgical removal was subsequently performed with extracorporeal circulation, followed by repair of the right ventricular wall. The postoperative course was uneventful, and the patient was discharged on postoperative day 10 without residual foreign bodies or structural cardiac abnormalities. CT = computed tomography, ECG = electrocardiogram, IVC = inferior vena cava, PMMA = polymethylmethacrylate, PVP = percutaneous vertebroplasty.

Figure 2 summarizes the sequence of the PVP procedure, symptom onset, diagnostic evaluation, surgical management, and postoperative recovery.

### 2.4. Management

Preoperative transesophageal echocardiography ruled out valvular involvement and newly developed pericardial effusion. Although the patient remained hemodynamically stable, the intracardiac cement fragment had a long, rigid configuration and was suspected to be penetrating the right ventricular wall. Because further migration could result in progressive perforation, malignant arrhythmia, or cardiac tamponade, surgical removal with extracorporeal circulation was planned.

During surgery, a small amount of bloody pericardial effusion was observed upon opening the pericardium. Right atriotomy revealed a segment of bone cement extending from the right atrium to the right ventricle, penetrating the right ventricular wall. Another segment of bone cement was identified at the entrance to the IVC. Both fragments were successfully removed, and the right ventricular wall was repaired to prevent further complications.

### 2.5. Outcome

The patient’s postoperative course was uneventful, with no residual cardiovascular complications. She was discharged after 10 days of follow-up, during which echocardiographic evaluation showed no recurrence of foreign bodies or structural abnormalities.

## 3. Discussion

The patient in this case presented with palpitations and perioral numbness. Initially, empirical treatment was administered based on the suspicion of coronary artery disease, but imaging revealed linear echogenic structures in the right atrium, right ventricle, and IVC, suggesting the possibility of cardiac cement embolism (CCE). Ultimately, a detailed patient history led to the diagnosis of cardiac embolism due to bone cement migration following PVP. This case highlights the importance of considering CCE in patients who present with cardiovascular symptoms after PVP and underscores the need for timely imaging evaluation to avoid missed or incorrect diagnoses.

This patient was clinically high risk despite initially stable vital signs, normal electrocardiography, and normal troponin levels. The risk was determined by the mechanical features of the embolus rather than by early biochemical or hemodynamic abnormalities: the cement extended across multiple right-sided cardiac structures, penetrated the right ventricular wall, and was associated with bloody pericardial fluid at surgery. These findings indicated an imminent risk of further perforation, cardiac tamponade, arrhythmia, and sudden hemodynamic deterioration. Therefore, stable initial observations should not delay definitive management when imaging suggests intracardiac penetration.

The occurrence of cardiac bone cement embolism involves multiple factors. First, the anatomical characteristics of the vertebral venous system are a key factor. Since the vertebral venous plexus lacks venous valves, bone cement may flow retrogradely into the IVC, subsequently reaching the right atrium, right ventricle, and even the pulmonary circulation.^[3]^ Second, the fluid dynamics of the bone cement play an important role, with low-viscosity cement being more prone to leakage during injection and forming distal emboli.^[4]^ Finally, hemodynamic effects may cause the bone cement to displace immediately after surgery or over time, with larger cement particles potentially penetrating the heart wall, leading to cardiac tamponade, perforation, or life-threatening arrhythmias.^[5]^

The diagnosis of CCE can be challenging. First, patients’ symptoms are nonspecific and may only manifest as palpitations, chest tightness, chest pain, or even no obvious discomfort, leading to misdiagnosis as coronary artery disease or pulmonary embolism.^[6]^ Second, imaging is critical for the diagnosis of CCE, but a single diagnostic tool may have limitations. This patient was initially misdiagnosed with coronary artery disease, but was ultimately diagnosed through cardiac ultrasound, coronary angiography, and CT. Therefore, for patients with cardiovascular symptoms after PVP, a multimodal imaging approach combining cardiac ultrasound, CT, and coronary angiography is recommended to improve diagnostic accuracy.

The treatment strategy for CCE depends on the size and location of the bone cement embolus and whether it causes hemodynamic instability.^[7]^ For asymptomatic or mildly symptomatic patients with small emboli, conservative treatment with regular follow-up and anticoagulation therapy may be sufficient to prevent secondary thrombus formation. In some cases, catheter-based retrieval of foreign bodies may be attempted; however, due to the high hardness and irregular shape of bone cement, the intervention is technically challenging, and success rates are limited. When bone cement causes cardiac penetration, hemodynamic instability, or presents a risk of cardiac tamponade, surgical intervention remains the preferred choice.^[4]^ In this case, the bone cement had penetrated the right ventricular wall, posing a significant risk of complications. The patient underwent open-chest surgery with extracorporeal circulation, successfully removing the foreign body and repairing the ventricular wall, thus avoiding severe consequences.

To improve the long-term prognosis for patients with CCE, postoperative management and follow-up are crucial. First, routine imaging follow-up should be performed after PVP, with periodic X-rays, CT, or cardiac ultrasound recommended to detect bone cement leakage early. Second, controlling the volume and viscosity of the bone cement during surgery is essential to reduce the risk of leakage, and the use of low-viscosity bone cement should be minimized. Additionally, clinicians should remain vigilant, and if PVP patients experience unexplained palpitations, chest pain, or shortness of breath, early imaging evaluation should be performed to rule out CCE. For patients at high risk of venous reflux, intraoperative balloon occlusion techniques may be considered to reduce the likelihood of bone cement entering the venous system. This case highlights the importance of imaging follow-up after PVP and the need for comprehensive evaluation using various imaging modalities in similar cases. Timely identification of CCE and the adoption of appropriate treatment strategies can optimize patient outcomes.

## 4. Conclusion

This case report highlights a rare but serious complication of PVP, in which bone cement migrated and caused cardiac perforation. Surgeons and interventional radiologists should be vigilant about this possibility and ensure appropriate postoperative monitoring to improve patient outcomes.

## Author contributions

**Data curation:** Yan Dong, Zhikun Fu.

**Methodology:** Yan Dong, Yongfeng Zhu, Zhikun Fu, Yaping Xu, Xiangli Zhang.

**Writing—original draft:** Yan Dong, Yaping Xu.

**Writing—review & editing:** Xiangli Zhang.
